# Synthesis of High-Entropy Oxide Nanopowders with Different Crystal Structures by Electrical Explosion of Wires

**DOI:** 10.3390/nano15080571

**Published:** 2025-04-08

**Authors:** Shuai Liu, Kangqi Liu, Liangwen Qi, Lanjun Yang

**Affiliations:** 1 School of Electrical Engineering, Xi’an Jiaotong University, Xi’an 710049, China; 18813198242@163.com (K.L.); yanglj@mail.xjtu.edu.cn (L.Y.); 2 School of Mathematics and Physics, Lanzhou Jiaotong University, Lanzhou 730070, China; 18742509171@163.com

**Keywords:** electrical explosion, high-entropy oxides, crystal structure

## Abstract

High-entropy oxides are a new type of material that consists of five or more principal elements in an equimolar or nearly equimolar ratio. They have many excellent properties and are rapidly becoming a hotspot for the development of new high-performance materials. In this study, electrical explosion is used for the first time to synthesize high-entropy oxide nanopowders with different crystal structures. (FeCoNiCrCu)O is the rock salt structure, (FeCoNiCrTi)O is the spinel structure, and (CoNiTiCuZn)O contains the two phases. According to the TEM and EDS results, the distribution of the five metal elements in the electrical explosion products is comparatively homogeneous, and the particle size of the products is concentrated in 20–40 nm. Elements such as Ti are prone to the formation of spinel structure, and the element Cu is prone to the formation of rock salt structure. The study shows that the electrical explosion of wires is a new method for the synthesis of high-entropy oxide nanopowders.

## 1. Introduction

In recent years, with the rapid development of science and technology, it has been challenging for traditional materials to meet the demands of social development, and the need for new high-performance materials has become more urgent. In this process, researchers combine various materials with exceptional performance to create new or improved materials [1]. After being mixed, new materials that have been developed in this way can increase the components’ average qualities and possibly create new ones that differ significantly from those of the previous ones. In 2004, Prof. Yeh suggested using the concept of disordered multi-component systems to maximize constructive entropy and decrease the system’s Gibbs free energy, resulting in a system of mixtures with each component stable. These materials are referred to as high-entropy materials [2]. High-entropy materials are a new class of multi-materials that are made up of several elements in nearly equal proportions and have an ordered structure and disordered chemical composition [3,4,5]. High-entropy oxides (HEOs), a type of high-entropy material, exhibit exceptional qualities such as the “cocktail” effect, hysteresis diffusion effect, lattice distortion effect, and high entropy effect, which have excellent potential applications in a variety of fields [6,7,8,9,10].

Considered the pioneer of high-entropy oxides, ROST successfully synthesized high-entropy oxide (MgCoNiCuZn)O with a rock salt structure in 2015, utilizing five binary metal oxides [11]. Numerous high-entropy oxides have been created as a result of the deepening of related research, and their structures can be divided into four groups: rock salt [12], fluorite [13], spinel [14], and perovskite [15]. Among them, rock salt and spinel are the two most common metal high-entropy oxide structures, with rare earth elements typically including the remaining structures. The rock salt structure, also known as the NaCl structure, is a face-centered cubic ionic crystal structure. Rock salt HEOs have a high specific capacity even after numerous cycle tests, making them valuable electrode materials for lithium-ion batteries. This is crucial for the growth of the new energy industry [16]. The standard formula for spinel structure is AB_2_O_4_, and by “splicing” together four A-type and four B-type small units, the unit cell can be divided into eight small cubic units, respectively. The ratio of the tetrahedral gap to the octahedral gap number is 2:1.

DABROWA created high-entropy oxides (CoCrFeMnNi)_3_O_4_ with a single-phase spinel structure for the first time in 2018, using oxides of the elements Co, Cr, Fe, Mn, and Ni [17]. Spinel HEOs are widely used in supercapacitors because of their excellent electromagnetic and electrochemical properties [18,19]. In high-entropy oxides, different phase structures can be interconverted with each other, and multiple phase structures may also arise simultaneously [20].

The conventional methods for the synthesis of high-entropy oxides, such as solution combustion synthesis and solid-phase sintering [21], have high yields and low synthesis costs, but they also require a long time to react and produce products with large particle sizes and uneven distribution [22]. Compared with the traditional method of metal nanopowder synthesis, the electrical explosion of wires (EEW) method has the advantages of high energy conversion efficiency, no pollution, single-step synthesis and uniform particle size distribution of the resulting powder, and it is not easy to form agglomerates, which is used more and more in the creation of new materials [23,24].

By using pulse discharge technology, the electrical explosion method deposits energy into metal wires quickly. The powerful pulse current causes the metal conductor to change from a solid state to a liquid, then a gas, and finally to break down into the plasma state. The metal wire expands and explodes, causing the metal vapor to move quickly and react with the ambient gases (e.g., oxygen) to generate nanoparticles by rapid cooling. EEW is considered to be an effective method for the synthesis of nanomaterials due to its extremely high rate of heating (dT/dt~10^11^ K/s) and quenching (10^10^ K/s). During the discharge process, multiple eruptions of metal vapor are caused by the breaking of plasma channels that form between the discharge electrodes [25,26].

The average particle size of the explosion products and the uniform composition distribution are related to the value of the ambient gas pressure and superheat ratio in the electrical explosion. The average particle size of the synthesized product exhibits a monotonic decrease with increasing the superheat ratio of the metal wire and decreasing the ambient gas pressure. By increasing the capacitor voltage while decreasing the diameter of the wire, the wire deposits enough energy, and its superheat ratio also increases. Sufficient energy also promotes the full reaction between multiple wires, resulting in a more uniform distribution of elements [27,28].

For the synthesis of metal nanopowders and binary alloy nanopowders by the EEW, researchers have successfully synthesized a variety of metal nanopowders such as Al, Cu, Fe, Ni, and other alloys such as NiCu, AlCu, etc., by electrical explosion of metal wires under inert gas [29,30,31]. In the synthesis of nano high-entropy alloy materials by EEW, K. Suliz of Russia produced AlCrFeCuNi alloys with FCC and BCC phase structures by electrically exploding metal wires in an argon atmosphere [32]. Wu Jian of Xi’an Jiaotong University created high-entropy alloy nanoparticles of Fe_10_Co_25_Ni_34_Cu_23_Al_8_ with an FCC phase structure [33]. The successful synthesis of high-entropy alloy materials by the EEW method provides a foundation for the synthesis of high-entropy oxides.

For the synthesis of monometallic oxide materials by the EEW method, Kiyoshi Yatsui in Japan synthesized nanopowders of alumina and titanium dioxide under oxygen [34]. Copper wires were electrically exploded in the air by L. Égerházi to produce a mixture of powdered copper oxide and cuprous oxide [35]. Additionally, work has been performed to create MoS_2_-ZnO and γ-Fe_2_O_3_ powders by electrically exploding zinc and iron wires, respectively [36,37]. For binary metal oxides, scientists created ZnO/ZnFe_2_O_4_/Zn mixed powders by electrically exploding twisted zinc and iron wires in the oxygenated atmosphere [38]. Elena Glazkova electrically exploded two twisted wires to obtain the multi-component product of CuFe_2_O_4_/Cu_2_O/CuO [39]. Regarding the multi-majority high-entropy oxides, no pertinent research has been conducted by EEW. In addition, previous studies for the synthesis of oxide nanoproducts have been carried out in air or oxygenated atmosphere, and no experiments have been carried out in oxygen atmosphere for the combined electrical explosion aspect of multi-wires.

In this study, high-entropy oxide nanomaterials with different crystal structures are synthesized by electrical explosion of combining multiple metal wires in an oxygen atmosphere for the first time, and the results show that it provides a new idea for the synthesis of high-entropy oxides. The physical phase structure of the explosion products is determined using XRD, and the distribution of particle sizes and elements within the products is determined using TEM. Finally, based on the above results, high-entropy oxide nanoparticles with different structures are successfully produced.

## 2. Materials and Methods

### 2.1. Experimental Setup

**Figure 1:**
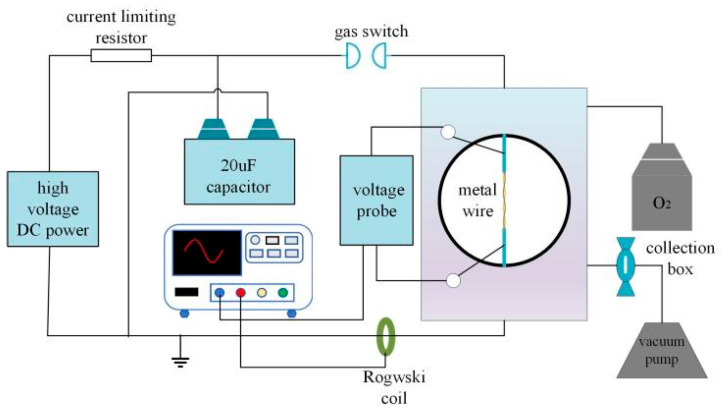
Schematic diagram of electrical explosion platform.

Figure 1 depicts the platform of electrical explosion utilized in this study. Before the experiment, a high-voltage DC power supply is used to charge the 20 μF capacitor. When it is charged to the required voltage, the charging power finishes charging, and a light signal triggers the gas switch, which discharges the capacitor to the wire. Before charging, all the initial air in the reaction chamber is pumped out with the vacuum pump and then filled with oxygen. After the explosion, black powder is observed to fill the experimental chamber. To collect the product, a PTFE hydrophobic filter membrane with a pore size of 0.1 μm is placed into the pumping line.

**Figure 2:**
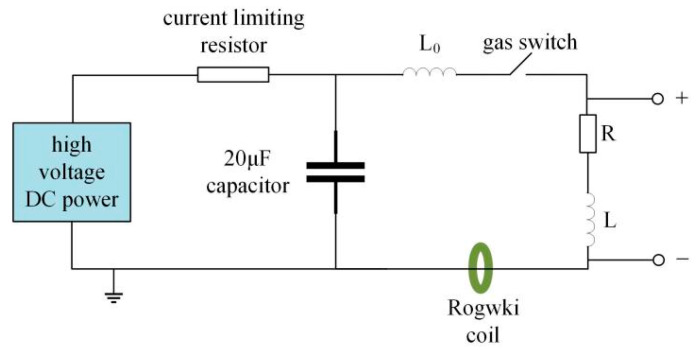
Equivalent circuit diagram of electrical explosion.

The experiment’s equivalent circuit in this study is depicted in Figure 2, where *R* is the wire’s resistance, which changes depending on the wire’s condition. *L* is the total inductance of the lead and wire, which has a value of 0.178 μH, while *L_0_* is the inductance of the loop. The voltage signal is measured with a high-voltage probe (North Star PVM-5, North Star High Voltage, Inc., Bainbridge Island, WA, USA), and the current signal is measured by a Rogowski coil (Pearson 4191, Pearson Electronics, Inc., Palo Alto, CA, USA). The voltage and current signals are recorded with an oscilloscope (Tektronix DPO 4102B, Tektronix, Inc., Beaverton, OR, USA). In the experiment, the measured voltage is the sum of the resistive and inductive voltages of the wire, which is given by the following expression:

(1) $$U=U_{R}+U_{L}=IR+L\frac{dI}{dt}$$

In the formula, *U* represents the voltage signal measured by the high voltage probe, and *U_R_* and *U_L_* are the wire’s resistive and inductive voltages, respectively. The energy deposited on the wire is

(2) $$E=\int U_{R}Idt$$

By considering the inductance *L* on the leads and extracting the resistive voltage waveform, the energy deposited on the wire is calculated.

### 2.2. Materials

Table 1 depicts the experimental conditions, where *l* and *d* are the wire’s length and diameter, respectively. Sample 1 is the combination of iron, cobalt, nickel-chromium alloy (Ni70Cr30), and copper wire explosion; Sample 2 is the combination of iron, cobalt, nickel-chromium alloy (Ni70Cr30), and titanium wire explosion; Sample 3 is the combination of cobalt, nickel, titanium, copper, and zinc wire explosion.

Wires used in experiments must be firmly twisted together to ensure complete contact and a favorable result [40]. This promotes a full fusion of the individual wires and the uniform distribution of the individual metal elements in the explosion products. *c* is the molar ratio of each element. It is evident that the separate elements’ atomic content ratios are quite near to each other, which is considered to be a favorable circumstance for the creation of high-entropy oxides. The elemental content distribution satisfies the condition that the individual major elements of the high-entropy oxides are between 5% and 35% [41].

The oxygen content of the ambient gas is important for the generation of oxide nanoproducts [42]. Elevated oxygen content promotes the production of metal oxidation and prevents the generation of other impurities. Therefore, oxygen is intentionally selected over air as the ambient gas in the wire electrical explosion experiments conducted in this study, which facilitates the synthesis of high-entropy oxide nanomaterials.

The electrical explosion of wires is carried out at 100 kPa oxygen pressure and sufficient capacitive voltage. The external electrical energy should be greater than the energy required for complete gasification of the metal wires (superheat ratio greater than 1), which enables enough energy to be deposited into the wire and react sufficiently with oxygen, leading to excellent high-entropy oxide nanoproducts.

The Gibbs free energy Δ*G* is frequently used to determine whether a single phase will form in high-entropy oxide systems:

(3) $$\Delta G=\Delta H_{mix}-T\Delta S$$

The smaller the enthalpy of mixing Δ*H_mix_*, the larger the entropy value *S*, the lower the Gibbs free energy of the system, and the more stable the system. The degree of chaos and disorder in a system is measured by entropy, one of the factors of thermodynamic systems that characterize the state of matter. The higher the degree of chaos and disorder, the higher the system’s entropy value. The entropy value of a compound may be exponentially related to the number of microstates Ω of the system [43]:

(4) S=klnΩ

where *k* is Boltzmann’s constant. According to Boltzmann’s equation, the above entropy value can also be transformed into

(5) $$S=-R\sum_{i=1}^{N}c_{i}\ln c_{i}$$

*R* is the gas molar constant (8.314 J·mol^−1^·k^−1^), *C_i_* represents the molar fraction of elements, and *N* is the total number of elements. When *N* is a constant value, the entropy value reaches its highest while the molar percentage of each major element is the same. The entropy value of a 5-principal-element equimolar high-entropy alloy is 1.61 *R*. When the entropy value of the alloy is less than *R*, it is a low-entropy alloy; between *R* and 1.5 *R*, it is a medium-entropy alloy; greater than 1.5 *R*, it can be called a high-entropy alloy. This definition can also be used for high-entropy oxides [44,45,46]. It should be noted that the disorder of both anions and cations influences the system’s entropy value in high-entropy oxides. Their entropy values are redefined as follows:

(6) $$S=-R\left[{\left(\sum_{i=1}^{N}c_{i}\ln c_{i}\right)}_{cation-site}+{\left(\sum_{j=1}^{N}c_{j}\ln c_{j}\right)}_{anion-site}\right]$$

Since the high-entropy oxides synthesized in this paper, the anions contain only oxygen ions and a small number of oxygen vacancies, the contribution of the anionic component to the overall entropy value is very small and negligible. Consequently, the atomic occupancy ratio of each major metal element in Table 1 is used to calculate the entropy value of the cation component. The entropy values of (FeCoNiCrCu)O are obtained as 1.534 *R*, (FeCoNiCrTi)O as 1.536 *R*, and (CoNiTiCuZn)O as 1.59 *R*. All of the above entropy values satisfy the conditions for forming high-entropy oxides.

The products obtained from the reaction are examined by X-ray diffractometer (Bruker D8 Advance, Bruker, Beilin, Germany, Cu Kα-radiation) to determine the structure of the products. The particle size distribution and elemental composition of the products are determined by TEM (FEI Talos F200X, Thermo Fisher Scientific, Waltham, MA, USA).

## 3. Results

### 3.1. EEW Results

**Figure 3:**
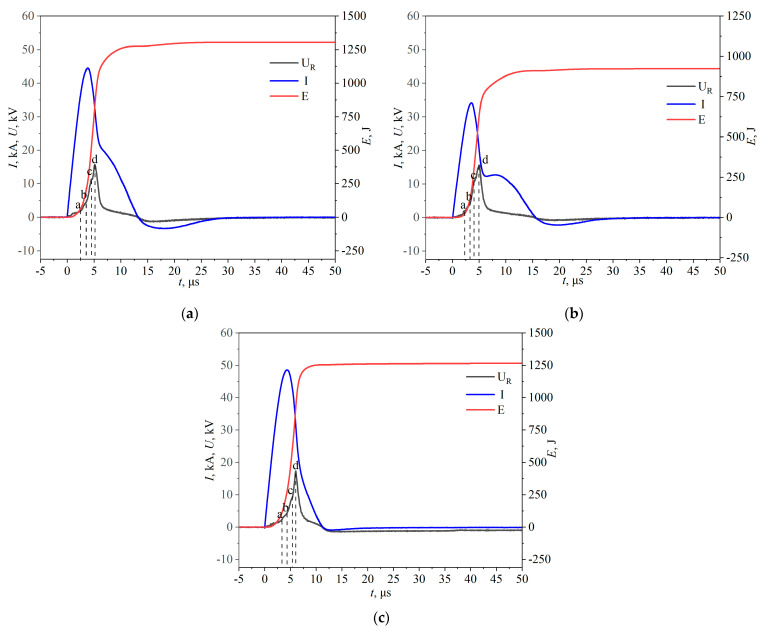
Electrical explosion voltage, current, and energy waveforms (**a**) (FeCoNiCrCu)O; (**b**) (FeCoNiCrTi)O; (**c**) (CoNiTiCuZn)O.

Figure 3 shows the resistive voltage, current waveforms, and energy deposition of a multi-wire joint electric explosion at an oxygen pressure of 100 kPa.

The a, b, c, and d points on the voltage curve are the obvious turning points; they represent several stages in the process of electrical explosion of the wire, respectively, for the solid-state heating (0–a), melting (a–b), liquefaction (b–c), and gasification (c–d). The moment of gasification ends (d), and its voltage reaches its maximum value. Following the moment of d, the explosion products swiftly spread to the surrounding medium with shock waves, undergo a chemical reaction with oxygen, and condense into nuclei. At the same time, the plasma with lower resistivity starts to form. After a period of time, an arc breakdown discharge is formed between the two high-voltage electrodes, and the energy is further deposited into the explosion products. This is evident in the stage of t > 10 μs, where the energy deposition is still increasing slowly and the voltage and current appear to be underdamped inverse waveforms. The secondary breakdown process of the plasma channel has an important contribution to the uniform distribution of the particle size of the explosion products.

The EEW waveform of (FeCoNiCrCu)O is shown in Figure 3a, where point 0–a is the solid-state heating stage. In this stage, energy is slowly injected into the metal wires, which have a low resistance and a slower rate of voltage rise. The melting process starts at 2.48 μs (point a), and during the melting stage, the resistivity of the individual wires gradually rises and the rate of energy injection quickens. The metal wires start to liquefy at 3.43 μs (point b). At this point, the resistivity of the copper and iron wires increases slowly, while the resistivity of the cobalt wires decreases slightly. Therefore, the overall resistance does not increase much, and the voltage increases more slowly during the liquefaction stage than during the melting stage. At this stage, the wires’ temperature is close to or even exceeds their boiling points, but they nevertheless remain liquid due to electromagnetic hoop shrinkage and the inertia effect, and their wires’ diameter barely changes. At 3.79 μs, the current reaches a maximum value of 44.54 kA; then the current begins to fall, the wire resistance rises, and its voltage still grows. At 4.5 μs (point c), the gasification of the wire begins, and the resistivity of each wire rises sharply. Under the influence of the skin effect, the outer layer of the wire gasifies first, and the diameter of the wire decreases, accompanied by the formation of a shock wave. In this stage, the resistance increases significantly, the wire voltage increases very quickly, and the current decreases at an accelerated rate. At 5.14 μs (point d), the voltage reaches a maximum value of 15.66 kV; at this time the energy deposited on the wire is 835 J. According to theoretical calculations, the four kinds of wire completely gasify the energy needed for 590.3 J; at this time, the superheat ratio of k = 1.41.

Based on this, it can be inferred that the metal wire is fully gasified at d, and its resistance reaches a maximum of R = 0.485 Ω. After point d, a low-resistance plasma discharge channel forms between the two high-voltage electrodes, resulting in a sharp decrease in the measured resistance voltage. The plasma channel is punctured, and the residual energy of the capacitor is further deposited into the explosion products with a final energy of 1304 J.

(FeCoNiCrTi)O is depicted in Figure 3b. At the liquefaction stage b (3.4 μs)–c (4.19 μs), the current reaches its maximum value at 3.55 μs, reaching a maximum value of 34.15 kA; at the gasification stage, the electrical explosion reaches its maximum value of the voltage at d (5.04 μs), reaching a maximum value of 15.07 kV. At this time, the energy deposited in the wire is 643 J; the theoretically calculated energy required for the complete vaporization of these four wires is 530.2 J. Therefore, the superheat ratio k = 1.2. After that, the metal vapors in the plasma state are penetrated by the residual energy, and an underdamped secondary discharge waveform is formed. At 7.88 μs, the secondary discharge current reaches 12.72 kA. Ultimately, the energy deposited in the explosion products is 922 J.

Figure 3c shows the situation of (CoNiTiCuZn)O. At 6.02 μs (point d), the voltage achieves its maximum value of 17.35 kV. The energy deposited in the wire at this moment is 920 J. The energy needed to completely vaporize the five wires is 647.6 J, according to theoretical estimates; at this point, the superheat ratio is k = 1.42. Subsequently, EEW still enters into the secondary discharge stage, and the plasma channel is broken down. Finally, the energy deposited in the explosion product is 1258 J.

The EEW waveform varies slightly due to the difference in physical properties of the wire and the capacitance voltage, but the general process is similar. The EEW process of Figure 3b is similar to that in Figure 3a, but slightly different. Since titanium wire requires less energy per unit volume to reach the melting point than copper wire, which makes the melting start at 2.3 μs (point a). This is earlier compared to the situation in (a). The time in the gasification stage is significantly longer compared with that in (a) because the capacitance voltage is lower, and the energy injection rate is slower. In comparison to the previous two situations, Figure 3c takes longer to reach the maximum value, and the peak value rises. This is due to the fact that more energy is required for five wires, and the resistivity increase is more obvious.

The voltage pulse appears only once in all three cases of electrical explosion, which indicates that these multiple wires are approximately synchronized electrical explosions [47]. The differences, such as the conductivity of the selected wires and thermophysical parameters, will not be particularly large. There is a secondary breakdown stage of the metal vapor in the plasma state in all three situations, which has significant effects on the explosion products.

### 3.2. XRD Data

**Figure 4:**
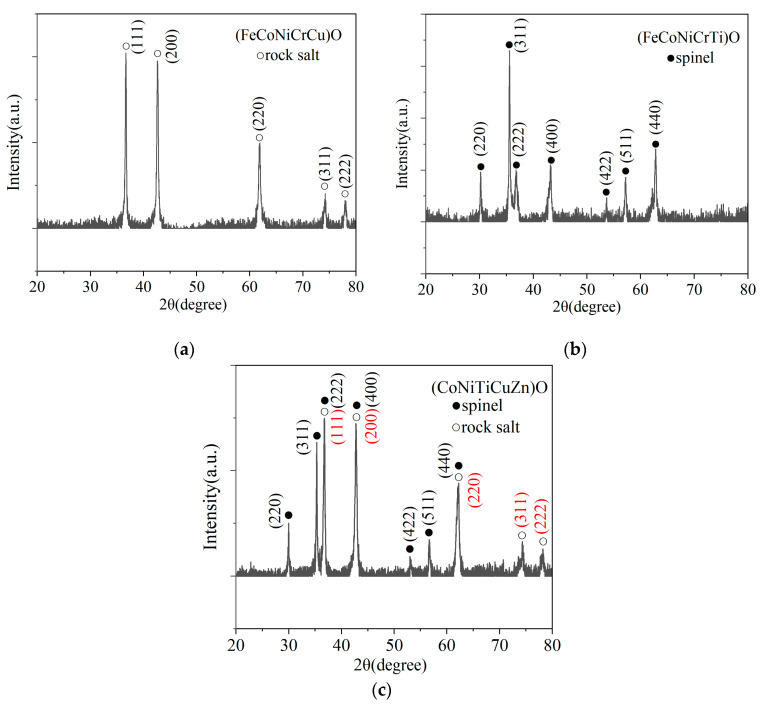
XRD pattern of explosion products (**a**) (FeCoNiCrCu)O; (**b**) (FeCoNiCrTi)O; (**c**) (CoNiTiCuZn)O.

The XRD data of the explosion products are shown in Figure 4, which shows that the three high-entropy oxides have different crystal structures.

The diffraction peaks of (FeCoNiCrCu)O at 36.7°, 42.6°, 61.8°, 74.2°, and 77.8° correspond to the crystal faces of (111), (200), (220), (311), and (222) of the rock salt structure, respectively. The space group is Fm-3m, and its calculated lattice parameter α ≈ 4.236 Å. The diffraction peaks of (FeCoNiCrTi)O at 30.2°, 35.6°, 36.9°, 43.2°, and 53.7° correspond to the crystal faces of (220), (311), (222), (400), and (422) of the cubic spinel structure, respectively. The space group is Fd-3m, and its calculated lattice parameter α ≈ 8.375 Å. The simultaneous presence of both rock salt phase and spinel phase crystal structures is evident in the (CoNiTiCuZn)O diffraction pattern. In Figure 4c, the red font is the crystal face of the rock salt structure, and the black font is the crystal face of the spinel structure. The diffraction peak at 36.8° contains both the (111) crystal face of the rock salt structure and the (222) crystal face of the spinel structure, which indicates that multiple phase structures can exist in high-entropy oxides.

Sharp diffraction peaks and narrow half-peak widths in all three diffraction patterns, along with the lack of heterogeneous peaks, suggest that the explosion products created a single-phase or multi-phase solid solution structure [48].

### 3.3. TEM Data

**Figure 5:**
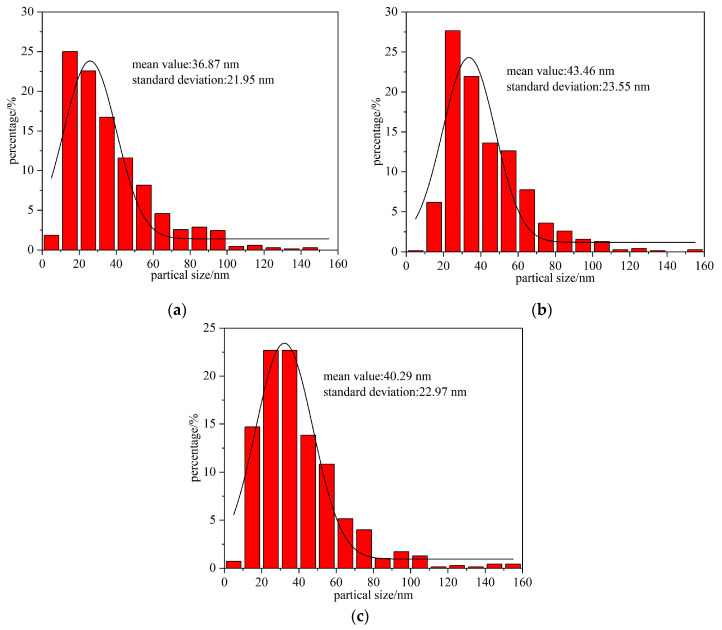
Particle size distribution of explosion products (**a**) (FeCoNiCrCu)O; (**b**) (FeCoNiCrTi)O; (**c**) (CoNiTiCuZn)O.

The explosion products are observed using TEM; 700 particles are selected by nano-measure software. Figure 5 shows the particle size distribution of the statistical products.

The average particle size of (FeCoNiCrCu)O is 36.87 nm, with a standard deviation of 21.95 nm; (FeCoNiCrTi)O is 43.46 nm, with a standard deviation of 23.55 nm; (CoNiTiCuZn)O is 40.29 nm, with a standard deviation of 22.97 nm.

It is evident that the particle size distribution of the products conforms to the log-normal distribution model proposed by scholars S.H. Park [49], and the values of the average particle size and the standard deviation of the products are determined by the probability density distribution function in the case of logarithmic distribution. The vast majority of the explosion products’ particle sizes are smaller than 100 nm, and the standard deviation is small, suggesting that the EEW method is a more effective means to synthesis high-entropy oxide nanomaterials.

The average particle size and standard deviation of (FeCoNiCrTi)O are the largest among the three, which may be attributed to the lower degree of reaction and the lower superheat coefficient in (FeCoNiCrTi)O. The superheating coefficients of (FeCoNiCrCu)O and (CoNiTiCuZn)O are nearly equal at the end of gasification, but the former’s average particle size is significantly smaller. This indicates that the energy produced by the secondary breakdown of the metal vapors in the plasma state further reduces the explosion products’ particle size [50]. The energy of the secondary breakdown is further deposited into the explosion products, reducing its average particle size. By increasing the superheating coefficient of the wire and by raising the energy of secondary breakdown, the mean value and standard deviation of the size of the electrical explosion products are reduced. This is consistent with the related study in the Introduction section [27,28].

**Figure 6:**
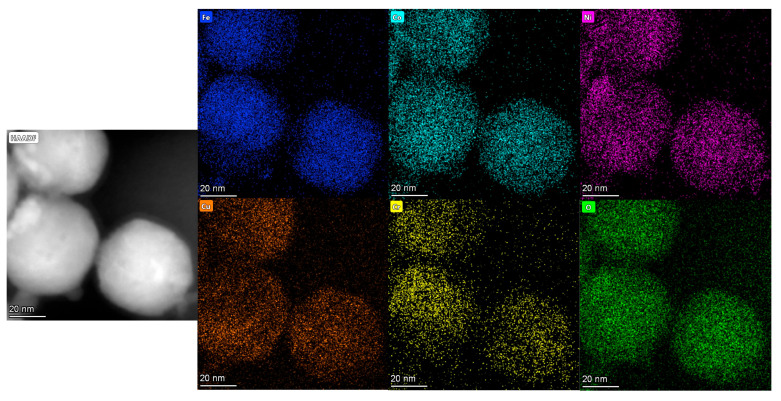
EDS results of (FeCoNiCrCu)O explosion products.

**Figure 7:**
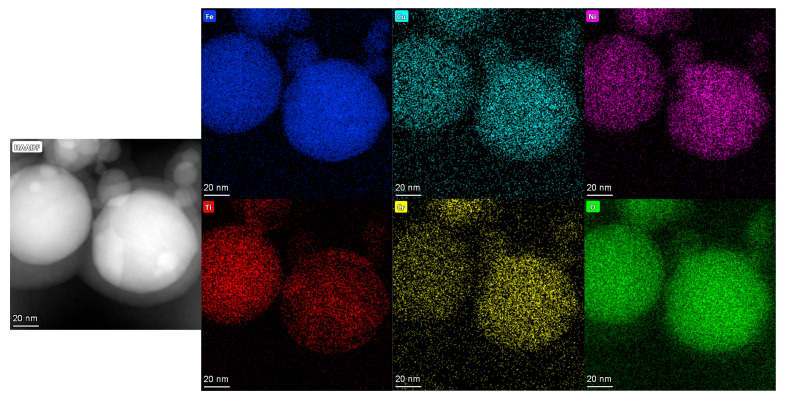
EDS results of (FeCoNiCrTi)O explosion products.

**Figure 8:**
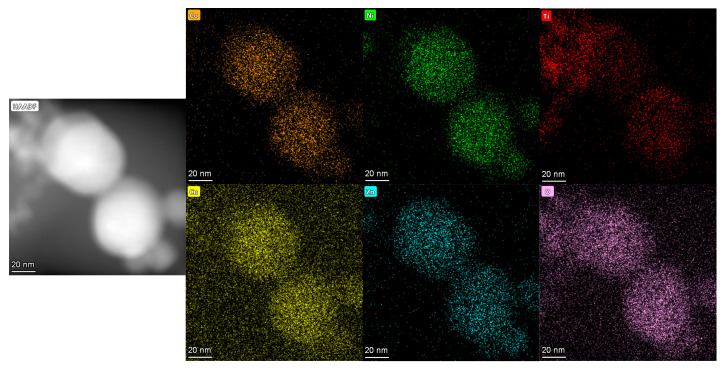
EDS results of (CoNiTiCuZn)O explosion products.

The results of EDS are shown in Figure 6, Figure 7 and Figure 8, and it is evident that the five principal metal elements and oxygen elements are uniformly distributed without serious segregation and distributed in the structure in a disordered manner.

The atomic percentages of constituent elements in the high-entropy oxides are quantitatively determined by EDS, with the corresponding results summarized in Table 2. Comparing the spinel structure of (FeCoNiCrTi)O and the rock salt phase of (FeCoNiCrCu)O, the content of oxygen element in the spinel structure is obviously increased. This is due to the fact that the number of anions and cations in the structure of the rock salt phase is relatively close to each other, while the structure of the spinel phase, in which the oxygen elements are arranged in the cubic closest packing arrangement, has a higher content of oxygen ions. The contents of major metal elements are similar to the theoretical calculations in Table 1.

Combining the above analytical tools, it can be shown that the EEW method is a significant method for the synthesis of high-entropy oxide nanoproducts.

## 4. Discussion

It has been demonstrated in related research that the crystal structures of high-entropy oxides are related to the synthesis method and the composition of their metal elements [51,52]. In this chapter, three other high-entropy oxides with different crystal structures—(FeCoNiCuZn)O, (FeCoNiTiZn)O, and (FeNiTiCuZn)O—are further synthesized using the EEW method in order to investigate in detail the impact of each component metal element on the crystal structure of high-entropy oxides.

### 4.1. Synthesis of Three Other HEOs with Different Crystal Structures by EEW

The experimental conditions for the EEW process are shown in Table 3. The meaning of its individual physical quantities is consistent with Table 1.

The entropy values of (FeCoNiCuZn)O, (FeCoNiTiZn)O, and (FeNiTiCuZn)O are 1.599 *R*, 1.593 *R*, and 1.594 *R*, respectively, according to the atomic occupancy ratio of each component metal in Table 3. They all satisfy the theoretical requirement that high-entropy oxides have an entropy value higher than 1.5 *R*.

**Figure 9:**
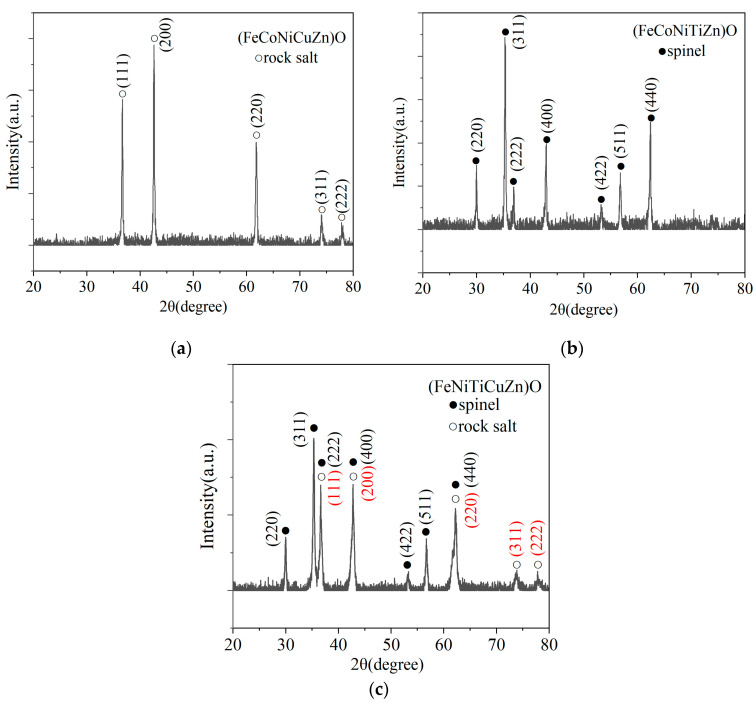
XRD pattern of explosion products (**a**) (FeCoNiCuZn)O; (**b**) (FeCoNiTiZn)O; (**c**) (FeNiTiCuZn)O.

The crystal structures of the three explosion products are examined using XRD, and Figure 9 displays their powder diffractogram.

(FeCoNiCuZn)O has a rock salt structure with calculated lattice parameter α ≈ 4.244 Å, and (FeCoNiTiZn)O has a spinel structure with calculated lattice parameter α ≈ 8.396 Å. Additionally, the simultaneous presence of both rock salt phase and spinel phase crystal structures is evident in the (FeNiTiCuZn)O diffraction pattern, just as (CoNiTiCuZn)O. The red font is the crystal face of the rock salt structure, and the black font is the crystal face of the spinel structure. In the powder diffractogram of (FeNiTiCuZn)O, the diffraction intensity at 36.7° is significantly higher than that of the (222) crystal face in the spinel structure because the (111) crystal face of the rock salt structure is also present in this diffraction peak.

### 4.2. Influence of Component Elements on Crystal Structures

In this study, a variety of high-entropy oxide nanoproducts with different crystal structures are successfully synthesized by electrical explosion for the first time. The experimental results are listed in Table 4.

It is evident from a comparison of the six high-entropy oxide nanoproducts produced in this paper using the EEW method that the kind of component metal elements will significantly impact the crystal structure of high-entropy oxides. For instance, it has been shown that the element Fe is easily combined with other metal elements to form a spinel ferrite structure in related studies [53].

As shown in Table 4, the rock salt structure of (FeCoNiCrCu)O and (FeCoNiCuZn)O compared to the spinel structure of (FeCoNiCrTi)O and (FeCoNiTiZn)O differ only in elemental Cu versus Ti. In addition, the dual-phase structure is observed in the HEOs containing both Cu and Ti elements. Rock salt structure is present in the HEOs with Cu elements, while spinel structure is present in the HEOs with Ti elements.

These results suggest that element Cu promotes the formation of rock salt structures in high-entropy oxides. This may be due to the fact that the CuO explosion products generated by the electrical explosion of copper wires can be gradually transformed into a rock salt phase structure at 22 KJ·mol^−1^ Gibbs free energy [54].

The element Ti promotes the formation of spinel structures in high-entropy oxides. This may be due to the electrical explosion of titanium wires in oxygen, which generates TiO_2_ nanoproducts that readily combine with other elements to form A_2_TiO_4_ (A = Co, Zn, Ni) products with a spinel structure [55].

It is also important to note that different synthesis methods have an effect on the crystal structure of high-entropy oxides. In the related study, (FeCoNiCuZn)O synthesized by solid-phase sintering method has a spinel structure [56]. This is caused by the transformation of the rock salt phase into a spinel phase after prolonged high temperature heating [57]. The presence of Fe element promotes the formation of spinel phases in HEOs.

In summary, the type of metal element has an important effect on the crystal structure of high-entropy oxides. Different synthesis methods may also produce phase changes in the crystal structure of HEOs. This result is important for the modulation of the crystal structure in the preparation of high-entropy oxides by electrical explosion. It is possible to transform the crystal structure by using different metal elements.

## 5. Conclusions

In this study, multiple wires are jointed for electrical explosion in an oxygen environment to obtain a variety of high-entropy oxides with different crystal structures for the first time. The explosion product properties are characterized using XRD, TEM, and EDS. The main conclusions are as follows:

1. The high-entropy oxide nanopowders with different crystal structures are successfully synthesized by EEW for the first time. (FeCoNiCrCu)O and (FeCoNiCuZn)O are rock salt structures, (FeCoNiCrTi)O and (FeCoNiTiZn)O are spinel structures, and (CoNiTiCuZn)O and (FeNiTiCuZn)O contain both rock salt and spinel phases;
2. The waveforms of the EEW process demonstrate that the electrical explosions of the individual wires are approximately synchronized, and the deposition energy is sufficient to completely evaporate the wires;
3. The TEM images demonstrate that the high-entropy oxides synthesized by the EEW method have a logarithmic distribution of particle sizes, with the vast majority of the particles below 100 nm and a small standard deviation. The distribution of each component element is uniform, and there is no enrichment;
4. The crystal structure of high-entropy oxides is significantly influenced by the kind of metallic elements. In the EEW synthesis method, the element Cu tends to form rock salt structure, while the elements Ti tend to form spinel structure. HEOs have a dual-phase structure in the presence of both Cu and Ti elements.

Based on the above results, this study successfully synthesized the nanopowders of high-entropy oxides with different crystal structures by the EEW method for the first time. The relationship between metal element kinds and the crystal structure of high-entropy oxides is also investigated. The joint electrical explosion of multiple metal wires might provide a new idea for the synthesis of high-entropy oxide nanomaterials.

## Figures and Tables

**Table 1:** EEW parameters.

Sample	Wire Material	*d*, mm	*l*, mm	*c*, %mol
1	Fe	0.3	44	24.45
	Co	0.3	44	26.2
	Ni70Cr30	0.3	44	Ni-17.4 Cr-7.45
	Cu	0.3	44	24.5
2	Fe	0.3	44	26.63
	Co	0.3	44	28.57
	Ni70Cr30	0.3	44	Ni-18.9 Cr-8.1
	Ti	0.3	44	17.8
3	Co	0.3	44	23.34
	Ni	0.3	44	23.44
	Ti	0.3	44	14.55
	Cu	0.3	44	21.79
	Zn	0.3	44	16.88

**Table 2:** The atomic percentage of each element in HEOs by EDS analysis.

Elements	(FeCoNiCrCu)O	Elements	(FeCoNiCrTi)O	Elements	(CoNiTiCuZn)O
O (At, %)	42.86	O (At, %)	62.56	O (At, %)	48.63
Fe (At, %)	13.29	Fe (At, %)	10.3	Co (At, %)	11.72
Co (At, %)	15.26	Co (At, %)	10.78	Ni (At, %)	12.1
Ni (At, %)	8.86	Ni (At, %)	7.08	Ti (At, %)	7.08
Cr (At, %)	3.48	Cr (At, %)	3.03	Cu (At, %)	12.42
Cu (At, %)	16.25	Ti (At, %)	6.25	Zn (At, %)	8.05

**Table 3:** EEW parameters.

Sample	Wire Material	*d*, mm	*l*, mm	*c*, %mol
1	Fe	0.3	44	21.74
	Co	0.3	44	23.33
	Ni	0.3	44	16.27
	Cu	0.3	44	21.78
	Zn	0.3	44	16.87
2	Fe	0.3	44	23.45
	Co	0.3	44	25.17
	Ni	0.3	44	17.55
	Ti	0.3	44	15.67
	Zn	0.3	44	18.2
3	Fe	0.3	44	22.1
	Ni	0.3	44	23.8
	Ti	0.3	44	14.8
	Cu	0.3	44	22.15
	Zn	0.3	44	17.15

**Table 4:** Crystal structure of high-entropy oxide nanoproducts.

Crystal Structure	High-Entropy Oxide Nanoproducts	
Rock-salt	(FeCoNiCr**Cu**)O	(FeCoNi**Cu**Zn)O
Spinel	(FeCoNiCr**Ti**)O	(FeCoNi**Ti**Zn)O
Dual-phase	(CoNi**TiCu**Zn)O	(FeNi**TiCu**Zn)O

## Data Availability

The data that support the findings of this study are available from the corresponding author upon reasonable request.
